# *ADRB2* Gene Polymorphism and Tocolytic Response to Hexoprenaline in Pregnant Russian Women with Threatened Preterm Labor

**DOI:** 10.3390/ph19071092

**Published:** 2026-07-16

**Authors:** Raisa A. Chilova, Ruslan E. Kazakov, Elvira V. Zhukova, Vasiliy B. Osadchev, Konstantin O. Akopov, Olesya S. Pobedinskaya, Maria V. Poznyak, Guzel F. Proklova, Evgeniya A. Svidinskaya, Oksana Yu. Gorbenko, Ekaterina O. Golubenko, Yuri V. Meksha

**Affiliations:** 1 Department of Obstetrics and Gynecology №1, Sechenov University, 119991 Moscow, Russiapoznyak_m_v@staff.sechenov.ru (M.V.P.);; 2 Department of Personalized Medicine and Clinical Pharmacogenetics, Scientific Centre for Expert Evaluation of Medicinal Products of the Ministry of Health of the Russian Federation, 127051 Moscow, Russia; 3 Women’s Clinic No.3, Yudin City Clinical Hospital, 115446 Moscow, Russia

**Keywords:** hexoprenaline, genotyping, tocolytic therapy, preterm labor, single-nucleotide polymorphisms, *ADRB2*, efficacy, safety

## Abstract

**Background:** Hexoprenaline, a widely used tocolytic, is a β2 adrenergic receptor agonist that when administered relaxes the smooth muscles in the uterus and cervix. The Gly16Arg polymorphism in the *ADRB2* gene is known to be associated with the risk of threatened preterm labor (TPL). Moreover, the Gly16Arg and Gln27Glu polymorphisms that form haplotypes can modulate receptor function, changing the pharmacological response to β2 agonists. **Aims:** This study aimed to investigate the effects of *ADRB2* gene polymorphisms on TPL and the response to tocolytic treatment with hexoprenaline in pregnant Russian women. **Materials and Methods:** This prospective case–control study on tocolytic therapy included 60 pregnant women with TPL who received tocolytic treatment with hexoprenaline and a control group consisting of 60 pregnant women not at risk of TPL. A polymerase chain reaction–restriction fragment length polymorphism (PCR-RFLP) analysis was conducted to determine the frequencies of Gly16Arg and Gln27Glu polymorphisms in the *ADRB2* gene in both groups. These data were compared with the TPL status and the tocolytic effect of hexoprenaline. **Results:** It is shown that carrying the *ADRB2* 16Arg allele is associated with decreased incidence of preterm birth (PTB), while the 16Gly/Gly genotype is related to an increased risk of PTB (OR = 2.444; CI95% = 1.167–5.121). The allele frequencies of the Gly16Arg and Gln27Glu polymorphisms in the *ADRB2* gene in healthy pregnant Russian women are consistent with those in the European population. Due to small subgroup sizes, tocolytic outcomes are presented descriptively without a powered statistical analysis. **Conclusions:** These findings suggest a preliminary association between the *ADRB2* 16Gly allele and the 16Gly/Gly genotype with an increased risk of PTB in pregnant Russian women. While carriers of the 16Arg allele may have a decreased risk, these pharmacogenetic insights require independent validation in larger cohorts before clinical recommendations can be formulated.

## 1. Introduction

The incidence of preterm births in different countries around the world varies from 5 to 18%. This level seems to not be decreasing and perinatal mortality continues to remain high, amounting to 28% [1]. Various medications are currently used to prevent preterm labor and prolong pregnancy [2]. Tocolytic therapy with beta_2_ agonists is mechanistically based on the stimulation of β_2_ adrenergic receptors located in the uterus and cervix, leading to the pronounced relaxation of smooth muscles. The β_2_ agonist hexoprenaline is used to induce acute, massive, or prolonged tocolysis, as appropriate. The mechanism of action of hexoprenaline consists of specific binding of the drug to β_2_ adrenergic receptors (as it interacts weakly with β_1_ adrenergic receptors), resulting in the activation of adenylate cyclase and intracellular cAMP production, important secondary messengers that trigger a cascade of processes changing the smooth muscle cell physiology in a regulatory manner.

There are three β-adrenoceptor genes in humans—*ADRB1*, *ADRB2*, and *ADRB3*. The *ADRB2* gene, encoding the β_2_ adrenergic receptor, is known to carry genetic variants influencing this receptor’s function. Two common missense mutations causing amino acid substitutions in codons 16 and 27 are of great interest: Gly16Arg (rs1042713 G > A) and Gln27Glu (rs1042714 C > G). Litonjua et al. (2010) highlighted the crucial role of the *ADRB2* gene in modulating smooth muscle tone and its overall pharmacogenetic significance [3]. Furthermore, evidence for *ADRB2* pharmacogenetics is well-documented in leading resources such as the Pharmacogenomics Knowledgebase (PharmGKB) and ClinPGx [4]. While the Gly16Arg polymorphism is widely known for altering receptor downregulation, the Gln27Glu polymorphism also plays a vital role in modulating the pharmacological response to β2 agonists by affecting receptor trafficking and resistance to downregulation [3,5]. These positions are located at the N-terminus of the protein molecule, outside the cell. Linkage disequilibrium is inherent in these polymorphisms and, as a result, they are inherited together in the form of stable haplotypes.

For the *ADRB2* gene, the frequency ratios of some haplotypes cannot be explained by simple gene drift [6]. The discovery of traces of natural selection on gene variability indirectly indicates the importance of its role in the pathogenesis of diseases. It has been reported that *ADRB2* gene polymorphism can be associated with autism, cerebral palsy, asthma, obesity, and metabolic syndrome, as well as preterm birth [6,7,8].

The number of receptors on the cell surface depends on the expression intensity and receptor internalization process. Different *ADRB2* genotypes are characterized by differences in both processes, which may impart a nonlinear dose-dependent effect [8].

Some studies have shown that *ADRB2* gene polymorphism affects the incidence of spontaneous preterm labor, which can be explained by modulation of the body’s susceptibility to the effects of β agonists [8,9,10,11,12,13,14]. It is known that homozygous carriage of the Arg/Arg16 allele of the *ADRB2* gene serves as a protective factor that reduces the probability of spontaneous preterm labor. Nevertheless, the association between the Gly16Arg polymorphic marker of the *ADRB2* gene and the likelihood of preterm birth cannot be considered to be resolved at present. There are few studies on *ADRB2* pharmacogenetics in relation to tocolytic therapy with β_2_ agonists [8,9,10,11,12,13,14]. It has been shown that pregnant women at risk of preterm birth carrying the 16Arg/Arg genotype of the *ADRB2* gene who are taking hexoprenaline have a more successful prolongation of pregnancy than carriers of the 16GlyGly or 16ArgGly genotypes [10].

While previous studies have explored these polymorphisms, data on the specific pharmacogenetic profile and its clinical implications in the Russian population remain scarce. The management of threatened preterm labor is increasingly shifting toward personalized approaches, driven by the highly variable efficacy and safety profiles of common tocolytics like β2 agonists and atosiban [15,16,17]. Despite established international guidelines [18], predicting individual patient responses to these drugs remains challenging in routine clinical practice [16]. Current pharmacogenetic evidence demonstrates that, beyond the *ADRB2* gene, other targets within the G-protein-coupled receptor signaling cascades (e.g., *GNAS, PPARGC1A*) significantly modulate both tocolytic efficacy and the risk of adverse cardiovascular events [19,20,21]. Additionally, given the multifactorial etiology of preterm birth, *ADRB2* polymorphisms likely interact with maternal systemic inflammatory pathways [22,23].

Our study aimed to investigate the effects of *ADRB2* gene polymorphisms on the risk of PTB and the tocolytic effect of hexoprenaline in pregnant Russian women.

## 2. Results 

### 2.1. Comparison of Main and Control Groups

The mean age of women who took hexoprenaline to prolong pregnancy was 31 ± 5.1 years (ranging from 19 to 44 years), while the mean age of women who reached term pregnancies was 30 ± 3.6 years (22 to 37 years) (*p* = 0.52). The samples were similar regarding height (study group, 167 ± 4.5 cm; control group, 166 ± 5.9 cm; *p* = 0.3901) and body weight (study group, 66 ± 9.6 kg; control group, 68 ± 11.5 kg; *p* = 0.5093). The proportion of obese patients was similar in both groups, at about 7%. Underweight (BMI < 18.5) was observed more often in pregnant women at risk of preterm birth (5 versus 1 in the control group) (*p* = 0.094). Thus, the threat of preterm birth in our study was independent of age, height, and obesity, but there was a tendency for higher risk of preterm birth in underweight participants.

The average gestational age among women with TPL who used tocolytic therapy was 38 ± 5.1 weeks. Among them, 21 women (35%) had preterm birth (PTB). The earliest births among the women with TPL were at 25 weeks, and the latest were at 40 weeks.

The average gestational age for women in the control group was 39 ± 0.6 weeks, with no cases of preterm birth among them. The earliest births in the control group were at 38 weeks, and the latest were at 41 weeks.

### 2.2. Genotyping Results

We performed genetic testing of pregnant women with TPL and controls for two single-nucleotide polymorphisms—Gly16Arg and Gln27Glu—of the *ADRB2* gene. Table 1 shows the frequencies of alleles and genotypes regarding the Gly16Arg polymorphism in the *ADRB2* gene in pregnant women with TPL and in controls, as well as the results of checking whether the observed genotype frequencies correspond to the theoretical ones (calculated using the Hardy–Weinberg equilibrium).

Table 2 shows the same information for the Gln27Glu polymorphism. Compliance with the Hardy–Weinberg equilibrium was observed for the control group for both polymorphisms, as well as for the Gly16Arg polymorphism in the TPL group. However, a deviation from the equilibrium was noted for the Gln27Glu polymorphism in the TPL group (*p* = 0.039), which is an expected finding in disease-associated cohorts. We found that the frequency of the 16Arg allele in the *ADRB2* gene in women in labor with TPL was 1.5 times lower than that in pregnant women in the control group (26% versus 40%; *p* = 0.028).

The comparison revealed that the frequencies of the genotypes of the Arg16Gly polymorphism in the *ADRB2* gene also differed significantly (*p* = 0.0499). At the same time, the frequency of the 16Arg/Arg genotype in pregnant women with TPL was 6.7% (4 women), which was 2 times lower that in the control group (13.3%; 8 women), although this difference was not significant (*p* = 0.833). In contrast, the “opposite” homozygous state of 16Gly/Gly was significantly more frequent in pregnant women with TPL: 33 (55%) versus 20 (33.4%) in controls (*p* = 0.027). Thus, we concluded that pregnant Russian women carrying the 16Gly allele, as well as the 16Gly/Gly genotype, have an increased risk of PTB (OR = 2.444; CI95% = 1.167–5.121); in contrast, carrying the 16Arg allele is associated with reduced risk of PTB (OR = 0.409; CI95% = 0.195–0.857).

Our study confirms what had already been discovered earlier by other researchers [13]; in particular, the 16Arg allele appears to play a protective role against the risk of preterm labor.

Comparing the frequencies of alleles and genotypes formed by the Gln27Glu polymorphism in the *ADRB2* gene, we found that, unlike the Gly16Arg polymorphism, it was not associated with a predisposition to the threat of preterm birth (*p* = 0.820 for alleles; *p* = 0.587 for genotypes).

Table 3 shows the allele frequencies of the *ADRB2* gene obtained in our study for pregnant Russian women with TPL and without pathology, compared with the literature data obtained in large samples representative of different ethnic groups.

In our study, out of 120 individuals tested, the 27Glu/Glu genotype corresponded to the 16Gly/Gly genotype in all cases (33 individuals), while the 16Arg/Arg genotype corresponded to the 27Gln/Gln genotype (12 individuals). Accordingly, we observe the inheritance of three stable haplotypes—namely, 16Arg + 27Gln, 16Gly + 27Gln, and 16Gly + 27Glu—whereas the 16Arg + 27Glu haplotype was absent in our study. The haplotype 16Arg + 27Glu is not known to occur in Europeans [6].

Table 4 and Table 5 provide the efficacy and safety indicators of hexoprenaline compared with the indicators for women in labor in the control group, depending on the *ADRB2* gene genotype. In Table 5, notation such as (I/I), (I/II), and (II/II) corresponds to the homozygous and heterozygous combinations of the haplotypes previously defined in the Caucasian population [5]. Data are presented as medians and ranges. The haplotype carrier genotyping results were interpreted based on information about carriage of the three abovementioned haplotypes in the Caucasian population. Only in three cases (two in the group taking hexoprenaline and one in the control group) was the newborn weight less than 2500 g. Preterm births occurred in 35% of the pregnant women taking hexoprenaline, while pregnancy was prolonged to more than 37 weeks in 65%. Cesarean section was performed in 31% of women in this group, of which 1/3 occurred earlier than 37 weeks.

Cesarean section was found to be significantly more frequent in the main group than in the control group (cumulatively for all genotypes).

PTB was observed in women with the 16Gly27Glu + 16Gly27Glu, 16Gly27Glu + 16Arg27Gln, and 16Gly27Gln + 16Gly27Gln genotypes at rates of 50%, 41%, and 62%, respectively. In these same subgroups, the requirement for Cesarean section was also notably high, at 66%, 18%, and 50%, respectively. Carriers of the 16Arg27Gln + 16Arg27Gln, 16Gly27Glu + 16Gly27Gln, and 16Arg27Gln + 16Gly27Gln genotypes did not have preterm births.

Adverse drug reactions to hexoprenaline occurred in 53% of pregnant women. Tachycardia was the most common, occurring in 47% of pregnant women receiving hexoprenaline. Unexpectedly, headache was also associated with taking this medication in 6% of pregnant women. The incidence of ADRs was significant in carriers of all *ADRB2* genotypes; the effect of gene polymorphism in this regard was insignificant.

As expected, birth weight and Apgar scores were lower in the TPL group, reflecting the result of prematurity itself rather than a pharmacogenetic response. The Ponderal index was not significantly different between the main and control groups. Dividing the group into subgroups by genotype reduced the sample size; therefore, the effects of genotypes on perinatal outcomes could not be identified.

## 3. Discussion

Our study shows that hexoprenaline is a reasonably effective tocolytic. In 65% of pregnant women taking hexoprenaline, pregnancy was prolonged to at least 37 weeks. Only one case of very early labor (25th week) could not be avoided. Moreover, in pregnant Russian women with threatened preterm labor, the frequencies of the minor alleles 16Arg and 27Glu were 26% and 50%, respectively; meanwhile, in the control group, these frequencies were 40% and 43%, respectively. These allele frequencies in the group of Russian healthy pregnant women correspond to typical values for the European population (39% for *16Arg* and 41% for *27Glu*) [5].

We also found that, in pregnant Russian women with threatened preterm labor, the frequency of the 16Arg allele was 1.5 times lower (*p* = 0.028) and the frequency of the 16Gly/Gly genotype was 1.65 times higher (*p* = 0.027) than those in the control group.

In pregnant Russian women, carrying the 16Gly allele, as well as the 16Gly/Gly genotype (OR = 2.444; CI95% = 1.167–5.121), was associated with an increased risk of preterm labor, while carrying the 16Arg allele was associated with a decreased risk of preterm labor (OR = 0.409; CI95% = 0.195–0.857).

Our findings regarding the protective role of the 16Arg allele and the risk associated with the 16Gly/Gly genotype align with recent updates in obstetric pharmacogenetics [15,22]. Recent comparative studies of β agonists emphasize that the clinical utility of tocolytics is frequently limited by maternal adverse reactions, highlighting the need for genetic risk stratification [18,20]. For instance, recent studies on tocolytic agents indicate that variations in β2 adrenergic receptors and associated signaling genes can significantly alter drug tolerance and efficacy [19,21,24]. As the paradigm shifts toward precision medicine in obstetrics, integrating *ADRB2* genotyping into clinical algorithms could minimize adverse events and optimize pregnancy prolongation strategies [15,24].

Our findings indicate that in pregnant Russian women, genetic factors—particularly the Arg16Gly polymorphism in the *ADRB2* gene—influence the risk of threatened preterm labor. Therefore, we suggest that identification of such genetic markers may eventually assist clinicians in risk stratification. However, independent validation in larger cohorts is strictly required before robust clinical recommendations can be formulated.

Our data add further evidence regarding the concept of personalized medicine, which seeks to identify the correct dose of the right drug for the right patient at the right time. Typically, the individualization of therapy is based on the pharmacogenomic makeup of the individual and environmental factors that alter their disposition and response with respect to the considered drug(s) [25].

We believe that the adoption of pharmacogenetic testing in obstetric care—especially for women at risk of developing TPL—is dependent on evidence that is analytically and clinically valid, supporting clinical utility. Our results show that the identification of pregnant women carrying the 16Gly allele and the 16Gly/Gly genotype who are at increased risk of preterm labor may provide valuable prognostic information, thus helping to better manage and potentially reduce maternal and neonatal risks. In contrast, carriers of the 16Arg allele appear to have a reduced risk of preterm labor.

## 4. Materials and Methods

The work was carried out at Sechenov University in Moscow. Before the start of the study, the study protocol and informed consent form were reviewed and approved by the local ethics committee (protocol numbers 16–19, dated 4 December 2019). All participants signed an informed consent form.

This prospective case–control study on tocolytic therapy included 60 pregnant women with threatened preterm labor (TPL) who received tocolytic treatment with hexoprenaline, constituting the main group. The control group consisted of 60 pregnant women who were not at risk of preterm labor, gave birth at >37 weeks, and received no tocolytic therapy.

Patients for the study were selected according to the following inclusion/exclusion criteria.

Inclusion criteria: Pregnant women with a single fetus, from 20 to 36 weeks gestation, age ≥ 18 years, the presence of regular (at least 2 within 10 min) contractions at 22–36 weeks gestation, cervical shortening <2.5 cm according to ultrasound with a vaginal transducer (Voluson E8; GE Healthcare, Chicago, IL, USA) and/or dynamic cervical changes (shortening and smoothing according to vaginal examination) and opening of the uterine cervix less than 3 cm, tocolytic therapy with the β_2_ adrenergic agonist hexoprenaline (Ginipral (Takeda Austria GmbH, Linz, Austria) was used), of Slavic descent for at least three generations, and informed consent to participate in the study.

Exclusion criteria: Multiple pregnancies, pregnancy after assisted reproductive technology (ART), premature rupture of fetal membranes, uterine malformations, significant fetal malformations, severe preeclampsia, pregnant women on immunosuppressive therapy (due to organ transplants or activation of autoimmune disease), acute infectious disease or activation of chronic infection with a temperature above 37.5 °C, severe extragenital diseases, and identified contraindications to hexoprenaline (hypersensitivity, primarily associated with bronchial asthma or hypersensitivity to sulfites; premature placental abruption; uterine bleeding; endometrial infections; cardiovascular diseases with tachyarrhythmia; myocarditis; heart defects; cardiomyopathy; coronary heart disease; arterial hypertension; severe liver and kidney diseases; thyrotoxicosis; angle-closure glaucoma; first trimester of pregnancy).

Tocolytic therapy with hexoprenaline was administered using an infusion system after dilution with isotonic sodium chloride solution to 10 mL. The given dose was 20 or 40 mg.

Information collected from pregnant women was related to the course of pregnancy, its outcome, and hexoprenaline tolerance (presence or absence of adverse reactions). The patients were also subjected to blood testing for genetic analysis. It should be noted that, in routine Russian clinical practice, calcium channel blockers such as verapamil are often used to manage cardiovascular adverse reactions to hexoprenaline; such concomitant medications were recorded.

The efficacy of hexoprenaline therapy was assessed according to the duration of pregnancy prolongation (>37 weeks).

Safety assessment included the monitoring and recording of adverse events throughout the study by assessing clinical and physical data, blood chemistry, general blood and urine tests, and maternal and fetal instrumental examination parameters. Only parameters showing clinical relevance or significant deviations—including cardiovascular responses (e.g., tachycardia) and neurological responses (e.g., headache)—are reported in the final analysis, as other parameters showed no significant fluctuations.

During this study, the absence of adverse drug reactions (ADRs) while evaluating the causal relationship with the study drug (WHO scale) was considered as a treatment safety criterion.

The Naranjo scale was used to assess the degree of reliability of the causal association between development of ADRs and the use of the drug in our work.

Genetic analysis was carried out at the Department of Personalized Medicine and Clinical Pharmacogenetics, Scientific Centre for Expert Evaluation of Medicinal Products.

Venous blood sampling for genetic testing was performed under clinical conditions in vacuum tubes for hematological studies with ethylenediaminetetraacetic acid (EDTA-K2) as an anticoagulant component (Becton, Dickinson and Company, Franklin Lakes, NJ, USA). Isolation of genomic DNA was carried out using a set of reagents for DNA extraction from the clinical AmpliPrime DNA-Sorb-V kit (Central Research Institute of Epidemiology, Rospotrebnadzor, Moscow, Russia).

Genotyping was performed via PCR-RFLP using a T100 thermal cycler (Bio-Rad Laboratories, Hercules, CA, USA) followed by electrophoresis. The following primers (Evrogen, Moscow, Russia) were used to amplify a gene fragment containing both polymorphisms:

- Direct: 5′-CCTTCTTGCTGGCACCCCAT-3′;
- Reverse: 5′-GGAAGTCCAAAACTCGCACCA-3′.

The amplification product had a total length of 308 bp.

For restriction enzyme digestion:

- The 16Gly allele was cleaved with the restriction endonuclease Bsp19 I (SibEnzyme, Novosibirsk, Russia), forming 291 bp and 17 bp fragments, while the 16Arg allele was not cleaved.
- The 27Gln allele was cleaved by the BstV1 I restriction endonuclease (SibEnzyme, Novosibirsk, Russia), forming 246 bp and 62 bp fragments, while the 27Glu allele was not cleaved.

Electrophoresis was conducted using a 12% polyacrylamide gel stained with ethidium bromide (Sigma-Aldrich, St. Louis, MO, USA), and visualized using a transilluminator and a Gel Doc EZ gel documentation system (Bio-Rad Laboratories, Hercules, CA, USA). To ensure the accuracy and reliability of the technique, the results were verified through selective sequencing of the amplification product.

The obtained data were statistically processed using the SPSS Statistics version 22 (IBM Corp., Armonk, NY, USA) software package. Cases with missing data were excluded in a pairwise manner. Two-sided testing was employed for all major comparisons. Descriptive statistics, Mann–Whitney U test, and Pearson’s chi-squared test were used. Odds ratios (ORs) were calculated with 95% Confidence Intervals (CIs). The significance level was set as α ≤ 5%, which is generally accepted in biomedical research.

## 5. Conclusions

Our study addresses a clinically relevant topic in obstetric pharmacogenetics, adding specific data for the Russian population. However, there are several important limitations. First, the total sample size of 120 participants (60 cases and 60 controls) is critically low for a genetic association study. Stratification by genotype combinations resulted in very small subgroups (e.g., 3 to 5 individuals), precluding statistically powered subgroup analyses. Second, methodological inferences regarding haplotypes were based on the previous Caucasian literature and databases (ClinPGx) [4,5], rather than derived from empirical phase analyses. Third, our definition of tocolytic efficacy did not fully account for all confounding factors, such as exact gestational age at the time of treatment, subclinical infections, cervical length, inflammatory biomarkers, and concomitant medications (e.g., verapamil). Finally, the control group consisted of healthy pregnancies without baseline risk or treatment; thus, the comparison measures the difference between a treated pathological pregnancy and a healthy one, rather than isolated pharmacogenetic variability in response.

## Figures and Tables

**Table 1:** Correspondence between observed frequencies of the genotypes of the Arg16Gly polymorphism in the *ADRB2* gene and theoretical frequencies calculated based on the Hardy–Weinberg equilibrium.

Allele/Genotype	Allele Frequencies/Number of Genotype Carriers	Number of Genotypes Calculated	χ^2^	*p*
Women in labor with threatened pregnancy who took hexoprenaline				
16Arg	0.26			
16Gly	0.74			
16Arg/Arg	4	4.0	0.002	0.967
16Arg/Gly	23	23.1		
16Gly/Gly	33	32.9		
Women in labor with normal pregnancy				
16Arg	0.40			
16Gly	0.60			
16Arg/Arg	8	9.6	0.741	0.389
16Arg/Gly	32	28.8		
16Gly/Gly	20	21.6		

**Table 2:** Correspondence between observed frequencies of the genotypes of the Gln27Glu polymorphism in the *ADRB2* gene and theoretical frequencies calculated based on the Hardy–Weinberg equilibrium.

Allele/Genotype	Allele Frequencies/Number of Genotype Carriers	Number of Genotypes Calculated	χ^2^	*p*
Women with threatened preterm labor who took hexoprenaline				
27Gln	0.50			
27Glu	0.50			
27Gln/Gln	19	15.0	4.267	0.039
27Gln/Glu	22	30.0		
27Glu/Glu	19	15.0		
Women in labor with normal pregnancy				
27Gln	0.57			
27Glu	0.43			
27Gln/Gln	22	19.3	2.065	0.151
27Gln/Glu	24	29.5		
27Glu/Glu	14	11.3		

**Table 3:** Frequencies of minor alleles of the Gly16Arg and Gln27Glu polymorphisms in the *ADRB2* gene in the studied samples, in comparison with those in other ethnic groups.

Sample	Frequencies of *ADRB2* Minor Alleles	
	16Arg Allele	27Glu Allele
Pregnant women with TPL (Russians)	26%	50%
Pregnant women in the control group (Russians)	40%	43%
Europe *	39%	41%
East Asia *	55%	7%
Africa *	52%	14%
World average *	48%	20%

* Data on ethnic variations in genotype frequencies of polymorphisms were taken from https://www.ensembl.org (accessed on 15 May 2026).

**Table 4:** Parameters of women in labor associated with the use of hexoprenaline compared with those in controls, depending on the *ADRB2* genotype.

Maternal *ADRB2* Genotype (Haplotype)	Group	*N*	Cesarean Section, *n* (%)	Preterm Birth (PTB), *n* (%)	Adverse Reactions to Hexoprenaline, %
16Gly27Glu + 16Gly27Glu (I/I)	TPL	18	12 (66%)	9 (50%)	68% (57% tachycardia; 11% headache)
	Control	14	0	0	-
16Gly27Glu + 16Arg27Gln (I/II)	TPL	17	3 (18%)	7 (41%)	53% (tachycardia)
	Control	21	0	0	-
16Arg27Gln + 16Arg27Gln (II/II)	TPL	5	0	0	50% (tachycardia)
	Control	8	1 (12.5%)	0	-
16Gly27Glu + 16Gly27Gln (I/III)	TPL	4	0	0	40% (tachycardia)
	Control	13	0	0	-
16Arg27Gln + 16Gly27Gln (II/III)	TPL	8	0	0	33% (headache)
	Control	11	0	0	-
16Gly27Gln + 16Gly27Gln (III/III)	TPL	8	4 (50%)	5 (62%)	44% (tachycardia)
	Control	3	0	0	-

Cesarean section was significantly more frequent in the main (TPL) group overall. Note: Roman numerals correspond to haplotypes identified in the Caucasian population: I = 16Gly27Glu; II = 16Arg27Gln; III = 16Gly27Gln. The 16Arg27Glu haplotype is considered absent in this population.

**Table 5:** Indicators of perinatal outcomes in women in labor in the main and control groups, depending on the *ADRB2* genotype.

Maternal Genotype	Group	*N*	PTB, *n* (%)	Birth Weight, g Median (Range)	Birth Length, cm Median (Range)	Apgar 1 min Median (Range)	Apgar 5 min Median (Range)
16Gly27Glu + 16Gly27Glu (I/I)	TPL	18	9 (50%)	2975 (2800–3570)	49.5 (49–51)	8 (7.5–8)	8 (8–9)
	Control	14	0	3355 (3141–3745)	50.5 (49.5–52)	8 (7–8)	8 (7–9)
16Gly27Glu + 16Arg27Gln (I/II)	TPL	17	7 (41%)	3370 (2960–3400)	51 (50–51)	8 (7–8)	8 (8–8)
	Control	21	0	3180 (2940–3400)	50 (49–51)	8 (8–8)	9 (8–9)
16Arg27Gln + 16Arg27Gln (II/II)	TPL	5	0	3300 (3200–3400)	50 (50–52)	8 (8–8)	8.5 (8–9)
	Control	8	0	3570 (3360–3670)	51 (50–53.5)	8 (8–8)	9 (8–9)
16Gly27Glu + 16Gly27Gln (I/III)	TPL	4	0	3530 (3250–3530)	53 ± 0.5	8 (8–8)	8 (8–8)
	Control	3	0	3250 (3200–3300)	50 ± 0.5	8 (8–8)	9 (8–9)
16Arg27Gln + 16Gly27Gln (II/III)	TPL	8	0	3285 (2900–3670)	52.5 (51–54)	8 (8–8)	8 (8–8)
	Control	11	0	3140 (3040–3340)	50 (50–50)	8 (7–8)	9 (8–9)
16Gly27Gln + 16Gly27Gln (III/III)	TPL	8	5 (62%)	2770 (2220–3030)	49.5 (47.5–51)	7 (6–7)	8 (7–8)
	Control	3	0	3250 (2870–3250)	53 (48–53)	8	9 (8–9)

Note: Data are presented as median (range). Statistical calculations (two-sided Mann–Whitney U test) were not performed when the number of newborns in either comparative subgroup was ≤5 (e.g., the II/II subgroup) due to insufficient statistical power for non-parametric testing. Haplotype notation: I = 16Gly27Glu; II = 16Arg27Gln; III = 16Gly27Gln.

## Data Availability

The original contributions presented in this study are included in the article. Further inquiries can be directed to the corresponding author.
